# User-centered design and usability testing of RxMAGIC: a prescription management and general inventory control system for free clinic dispensaries

**DOI:** 10.1186/s12913-018-3517-8

**Published:** 2018-09-10

**Authors:** Arielle M. Fisher, Timothy M. Mtonga, Jeremy U. Espino, Lauren J. Jonkman, Sharon E. Connor, Nickie K. Cappella, Gerald P. Douglas

**Affiliations:** 1 Center for Health Informatics for the Underserved, Department of Biomedical Informatics, School of Medicine, University of Pittsburgh, Pittsburgh, PA USA; 2 University of Pittsburgh School of Pharmacy, Pittsburgh, PA USA

**Keywords:** Health information interoperability, Health level seven, RxNorm; pharmacy information system, Electronic health records, Vulnerable populations, Evaluation studies, Medical informatics applications, Medical dispensaries, Free clinics

## Abstract

**Background:**

To address challenges related to medication management in underserved settings, we developed a system for Prescription Management And General Inventory Control, or RxMAGIC, in collaboration with the Birmingham Free Clinic in Pittsburgh, Pennsylvania. RxMAGIC is an interoperable, web-based medication management system designed to standardize and streamline the dispensing practice and improve inventory control in a free clinic setting. This manuscript describes the processes used to design, develop, and deploy RxMAGIC.

**Methods:**

We transformed data from previously performed mixed-methods needs assessment studies into functional user requirements using agile development methods. Requirements took the form of user stories that were prioritized to drive implementation of RxMAGIC as a web-application. A functional prototype was developed and tested to understand its perceived usefulness before developing a production system. Prior to deployment, we evaluated the usability of RxMAGIC with six users to diagnose potential interaction challenges that may be avoided through redesign. The results from this study were similarly prioritized and informed the final features of the production system.

**Results:**

We developed 45 user stories that acted as functional requirements to incrementally build RxMAGIC. Integrating with the electronic health record at the clinic was a requirement for deployment. We utilized health data standards to communicate with the existing order entry system; an outgoing electronic prescribing framework was leveraged to send prescription data to RxMAGIC. The results of the usability study were positive, with all tested features receiving a mean score of four or five (i.e. somewhat easy or easy, respectively) on a five-point Likert scale assessing ease of completion, thus demonstrating the system’s simplicity and high learnability. RxMAGIC was deployed at the clinic in October 2016 over a two-week period.

**Conclusions:**

We built RxMAGIC, an open-source, pharmacist-facing dispensary management information system that augments the pharmacist’s ability to efficiently deliver medication services in a free clinic setting. RxMAGIC provides electronic dispensing and automated inventory management and alerting capabilities. We deployed RxMAGIC at the Birmingham Free Clinic and measured its usability with potential users. In future work, we plan to continue to measure the impact of RxMAGIC on pharmacist efficiency and satisfaction.

**Electronic supplementary material:**

The online version of this article (10.1186/s12913-018-3517-8) contains supplementary material, which is available to authorized users.

## Background

Medication management is a complex and expensive continuum that covers all aspects of prescription medications, which are a critical component in healthcare delivery [1, 2]. Bell et al. model this continuum in five main phases: prescribing and ordering, order communication, dispensing, administering, and monitoring [3]. Each phase has high potential for both benefit and harm, especially in medically vulnerable populations and the low resource clinics that provide their care [1, 4]. These patient populations are often burdened with multi-morbidity requiring multiple medications, limited health literacy leading to inappropriate medication use, and cost-related medication non-adherence [2, 4]. These challenges, coupled with other preventable medication errors, may lead to worsening of disease, death, and increased healthcare costs [4, 5].

Appropriate medication management information technologies can reinforce each phase of this continuum. However, the effects of these technologies on medication management have not been adequately studied in nonprofit safety net clinics and health centers, which serve approximately 24 million people in the United States annually [6]. Pharmacists in these settings are often tasked with establishing a medication formulary, managing consumption and procurement, identifying cost-effective therapeutic alternatives, and educating patients on the importance of medication adherence. The challenging aspects of these tasks are exacerbated due to a lack of resources and the tools to facilitate effective management of those resources, such as automated systems to support drug procurement and distribution [6].

Free clinics may obtain drugs through a variety of channels, including the donation of drug samples from licensed practitioners, discounted bulk purchases, and Patient Assistance Programs (PAPs) [7]. PAPs are offered by many pharmaceutical companies and provide prescription medications for free or at a greatly reduced cost to those who cannot afford them. Many free clinics facilitate the application process for PAPs, which can be labor-intensive as eligibility requirements can vary across programs. Further, clinics are often responsible for receiving and dispensing medications to these patients on-site. Managing multiple, disparate inventory sources is a challenge unique to free clinics as they are typically stored independently.

Technology to facilitate aspects of medication management can play a significant information support role in these settings if designed to meet users’ needs and resource expectations. Existing medication management systems are generally designed for large hospital or community pharmacies, often as integrated or add-on modules to Electronic Health Records (EHR). These products may provide unnecessary functionality at a significant cost which is neither affordable or sustainable for a resource-limited clinic [8–10]. While stand-alone systems do exist, and may be less expensive, they can only do the tasks for which they are designed and are unable to exchange relevant health information with other systems in an enterprise organization [10].

Free clinics could benefit from a medication management system that incorporates characteristics of both integrated and stand-alone systems. That is, a problem-driven system that is uniquely designed to alleviate challenges associated with medication management in these settings. The system should have simple, limited functionality and require minimal resources to implement and sustain. Most importantly, the system should be capable of communicating with existing systems in the clinic to eliminate redundant work, such as similar data entry tasks to document patient care, order medications, and label prescriptions.

To this end, we built an open-source system for Prescription Management And General Inventory Control (RxMAGIC) that augments the pharmacist’s ability to efficiently deliver medication services in a free clinic setting. In previous publications, we describe workflow challenges encountered in a free clinic dispensary as typified by the Birmingham Free Clinic (BFC) [7, 11]. These publications report the results of a mixed-methods needs assessment study that were used to identify processes amenable to an informatics intervention and measure their relative impact on pharmacist efficiency [7, 11]. Our results concluded that dispensing and managing multiple inventory sources, both paper-based systems, were among the most inefficient and error prone processes [7].

In this manuscript, we introduce RxMAGIC and describe how it alleviates workflow challenges uncovered in a free clinic setting. We describe the user-centered design process employed to define and evaluate functional requirements, in addition to the health data standards required for system integration and data exchange.

## Methods

### Setting

This research was performed at the Birmingham Free Clinic (BFC) in Pittsburgh, PA, which is a non-federally-funded, walk-in health clinic in the city of Pittsburgh. Volunteer pharmacists and students work collaboratively with the medical team to provide primary care services to approximately 1900 patients annually. An on-site dispensary enables free access to prescription medications that are obtained through a variety of sources, including PAPs. The clinic employs a full-time staff person(s), typically a member of the AmeriCorps national service program, to manage the approximately 20% of the clinic’s patient population that are enrolled in PAPs. The AmeriCorps member oversees application cycles and facilitates reordering of medication to replenish a patient’s drug supply on a rolling three to four-month basis.

The BFC was founded under the auspices of the University of Pittsburgh Medical Center (UPMC) and is a fully implemented EHR site. UPMC donated EpicCare (Epic Systems Corporation, Wisconsin, USA), an ambulatory EHR module, to the BFC with the goal of standardizing clinical practice within their enterprise [12]. In this setting, EpicCare is used as a clinical documentation module (e.g. medical history, diagnoses, medication history) with Computerized Physician Order Entry (CPOE) and electronic prescribing, thus primarily serving the needs of the medical team.

Pharmacists at the BFC have access to the EHR for clinical work, however, due to resource constraints, there is no additional module that supports electronic dispensing, inventory management, or other processes specific to a dispensary workflow. They continue to utilize a paper-based workflow to dispense medications, track dispensation records, and manage inventory levels. While this paper-based system was inefficient prior to EHR implementation, its inefficient aspects were exacerbated post-EHR implementation due to redundant data entry tasks to maintain accuracy across both systems. Pharmacists want to leverage CPOE in the EHR to facilitate electronic dispensing and automated inventory control.

### Determination of functional requirements

We transformed data from the previously performed mixed-methods needs assessment study into functional user requirements [7, 11]. Requirements initially took the form of agile user stories, which are high-level statements describing desired software features and their benefits from an end-user perspective [13]. An example of a user story in this context is:

“As a pharmacist, I want to be notified when a medication item has fallen below a given threshold so that I can promptly add the item to the order sheet to avoid stock-outs.”

We grouped user stories into themes and prioritized them so that the highest priority items could be implemented first in the RxMAGIC prototype. These themes comprise those uncovered in our previous contextual inquiry [7], which were prioritized in such a way to address workflow challenges we measured to be the most inefficient in our time-motion study [11]. The staff at the BFC reviewed, modified, and validated the user stories prior to implementation.

Use cases were developed from collections of user stories and were accompanied by screen mockups for each desired interface. Screen mockups were developed with Mockingbird (https://gomockingbird.com/home) and shown to users and subject matter experts for concept validation.

### Determination of standards needed for integration

We studied the technology and standards needed to utilize the existing electronic prescribing framework at the clinic to send medication orders from the EHR to RxMAGIC. Standards are necessary to ensure that disparate systems can communicate with each other and have the same interpretation of the data that are transmitted between them. Ultimately, the use of standards is a benchmark indicating successful information technology implementation [14].

### Implementation of RxMAGIC

We implemented RxMAGIC as a web-application using agile development methods and the MoSCoW framework for feature prioritization. The MoSCoW framework classifies feature requirements in terms of “Must have,” “Should have,” “Could have,” and “Won’t have.” We utilized GitHub as a software versioning repository. The system was programmed using Ruby (v 2.2.3) on Rails (v 4.2.0) [15]. We used MySQL as a backend database and Mirth as a Health-Level-Seven (HL7) message router.

We built an initial functional prototype based on our user stories to test the viability and usefulness of RxMAGIC at BFC before developing a fully functional system. The prototype captured enough functional aspects of the desired system to provide a greater technical understanding of the problem and its potential users. We evaluated the prototype in the laboratory setting with potential users (i.e. pharmacy students) to understand its perceived usefulness. Users identified positive and negative features of the system in addition to suggestions for improvement. Following the initial implementation of the prototype we incrementally expanded functionality of RxMAGIC – conducting functional testing at each development iteration.

### Usability study

Upon completing the development of the production system, we evaluated the usability of RxMAGIC to understand how real users would interact with it in a simulated setting. The goal of this study was to facilitate identification of potential usability problems that may be avoided through informed redesign. The study protocol comprised think-aloud studies accompanied by screen recordings of user interactions with the system in addition to a usability survey.

Prior to the usability study, we piloted the study protocols and data collection tools. This was done to resolve any issues before the beginning of the real study. Data collected during the pilot study were not included in the usability analysis.

We used think-aloud protocols to gain a realistic understanding of how users navigate the system without any training on how to do so. The need for minimal training was a key requirement in this instance due to the high turnover of users at the BFC as a result of the volunteer-based model. Think-aloud studies require the user to verbalize their motions, thoughts, and challenges as they complete a collection of benchmark tasks within the system.

Each participant was asked to complete 11 representative tasks and rate its ease of completion on a five-point Likert scale. An average score was calculated for each task at the end of the study. Additional reactions and perceptions of the system were discussed with the participant following completion of each session and were documented for further consideration.

We used Kazaam Screencaster for Ubuntu (https://launchpad.net/kazam) to record on-screen action and audio into a video file. Each file was annotated using ChronoViz (http://chronoviz.com/) to identify common patterns amongst participant responses. These responses included descriptions of positive and negative features of the system, potential interaction problems, and suggestions for improvement. We aggregated these responses and compiled a prioritized list of system modifications to be implemented before deployment.

### System deployment

We deployed RxMAGIC in three phases: 1) configuration of the computers at the clinic administration office, 2) a physical stock count and data entry of all medications at the clinic, and 3) installation of both the inventory and dispensing modules at the clinic. Most users were trained on-site over the course of a two-week period following deployment using a combination of our training video and hands-on user training sessions. The training video is available at https://www.youtube.com/watch?v=BRMK-JLuYOg&t=202s.

## Results

### Determination of functional requirements

We developed and prioritized 45 user stories from the perspectives of three user groups: pharmacists (34), AmeriCorps (7), and physicians (4). User stories were grouped into eight implementation themes which include: 1) view and maintain inventory through a web-based browser, 2) use electronic dispensation to update drug counts in real time, 3) produce computer generated labels upon dispensation, 4) automatically alert pharmacists of new medication orders, 5) provide inventory alerts in real-time, 6) automatically generate daily dispensation record and enable PDF export, 7) provide medication reordering support for PAP applications, and 8) establish a user management framework. Prioritization was informed by the results from the previously performed time-motion study [11]. All user stories are summarized in Additional file 1.

As RxMAGIC is a pharmacist-facing application, we recognized that the user stories from the physician perspective describe many of the same user needs as the other two user groups. Additionally, as physicians were not expected to be users of the application, we prioritized pharmacist and AmeriCorps user stories to ensure the system meets the needs of our targeted users.

### Determination of standards needed for integration

To ensure a successful system adoption, RxMAGIC had to communicate with EpicCare to receive prescription data. To achieve this, we leveraged the pre-existing HL7 (version 2.3) outgoing messaging framework already used for electronic prescribing to facilitate RxMAGIC dispensing at the clinic. HL7 is a widely used messaging standard that provides a framework for the exchange, integration, sharing, and retrieval of electronic health information between disparate applications [16]. We implemented a feature in RxMAGIC wherein EpicCare sends prescription data to RxMAGIC using a unidirectional HL7 messaging connection; RxMAGIC does not send any data back to the EHR.

Amongst other fields, the HL7 message from EpicCare provides the National Drug Code (NDC) of the prescribed medication. We use this NDC identifier to map proprietary medication terms found in EpicCare to standard RxNorm terms that are used throughout RxMAGIC. RxNorm is a non-proprietary clinical drug nomenclature developed and maintained by the US National Library of Medicine that can be used to ensure a shared meaning of data between systems using different drug vocabularies (https://www.nlm.nih.gov/research/umls/rxnorm/).

We utilized the Lightweight Directory Access Protocol to enable users to log into RxMAGIC using their existing UPMC credentials. This was a functional requirement to operationalize RxMAGIC as it is a system within the UPMC enterprise.

### Implementation of RxMAGIC

RxMAGIC augments aspects of the multi-stage medication management continuum. These high-level processes include: electronic inventory tracking, order communication and interoperability, electronic dispensing, and alerting and reporting. We describe how RxMAGIC supports each of these processes below.

#### Electronic inventory tracking

**Fig. 1:**
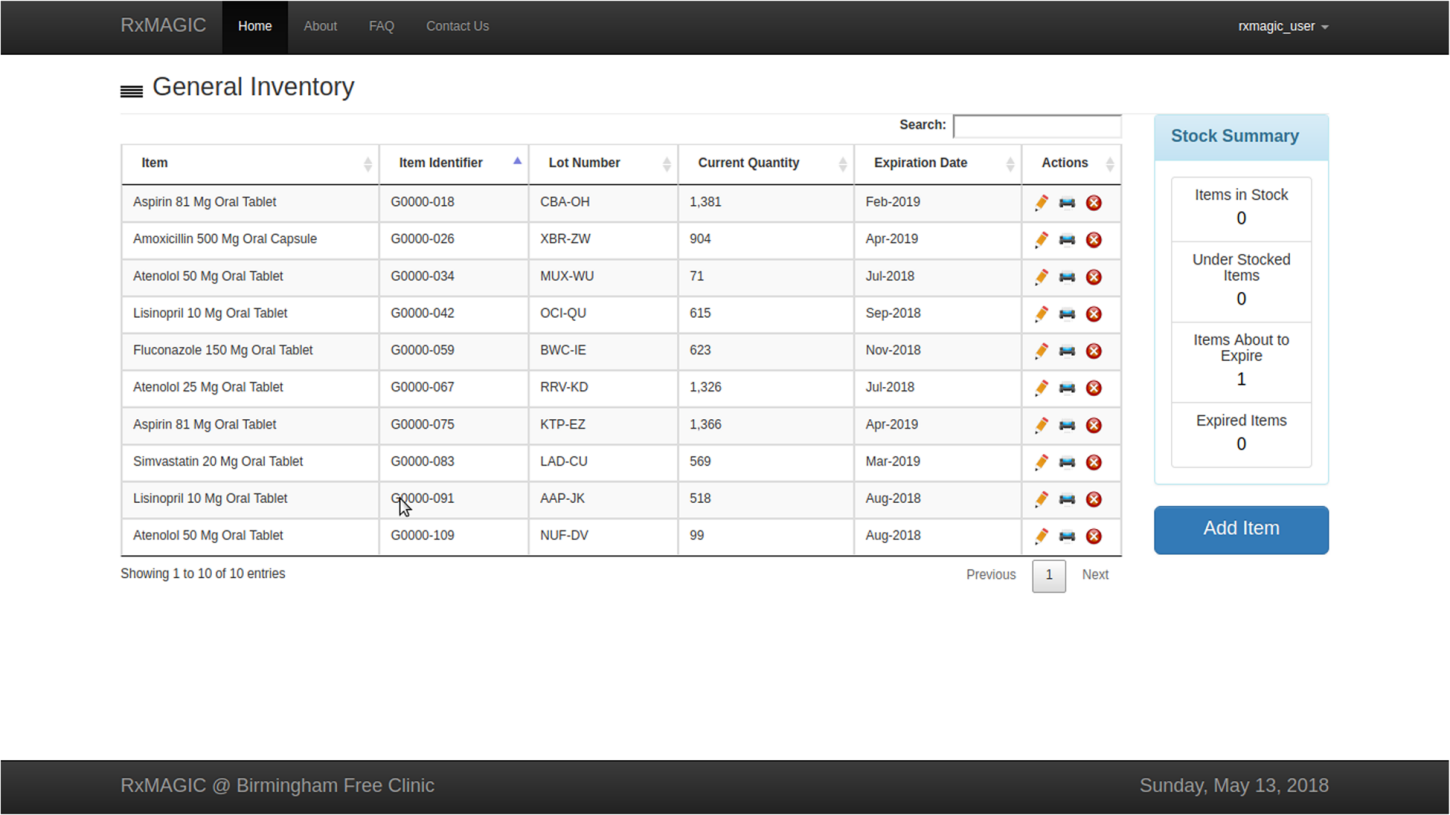
General inventory view. Users can add and manage existing inventory items in this view. The PAP inventory view is similar, although the ‘add item’ workflow is different as described

RxMAGIC provides automated inventory control and visibility by tightly coupling each medication item to a unique barcode. When new medications arrive, an AmeriCorps member logs their receipt by entering medication details (i.e. name, expiration date, manufacturer, lot number, and received quantity) in RxMAGIC, which creates a unique barcode label that is affixed to each medication item. Users can view the complete inventory and medication details such as lot number, current quantity, and expiration date as shown in Fig. 1.

The inventory screen also includes a stock summary panel that describes low-inventory items, well-stocked items, items approaching expiration, and expired items (Fig. 1). When selected, these features provide a more granular understanding of inventory levels to facilitate efficient replenishing. Stock availability is determined by comparing the current drug quantity to a predetermined par level. A par level, or threshold, is the minimum level of inventory necessary to be on hand in the dispensary at any time. Par levels in RxMAGIC are customizable and can be created at any time within the application.

#### Electronic patient assistance program inventory tracking

As earlier discussed, RxMAGIC also manages the Patient Assistance Program (PAP) inventory. These medications are procured and dispensed to specific patients, therefore, the inventory entries are tightly coupled to the patient record. In addition to the documentation required for general inventory medicines, users are also required to enter a ‘date to reorder’ the medication to prompt staff to reorder ensuring the next supply. RxMAGIC supports this reordering process by providing users a configurable report of all medications approaching their reorder date.

#### Order communication and interoperability

RxMAGIC does not change the medication ordering process; physicians continue to place medication orders using the EHR. When a new order is placed, it is packaged as an HL7 message and electronically transmitted to RxMAGIC. Identifiable information in the HL7 message is used to create a unique patient record within RxMAGIC. If the data abstracted from the HL7 message matches an existing patient, then the prescriptions are associated to that patient’s record. A patient’s dispensation history can be viewed at any time within the application.

**Fig. 2:**
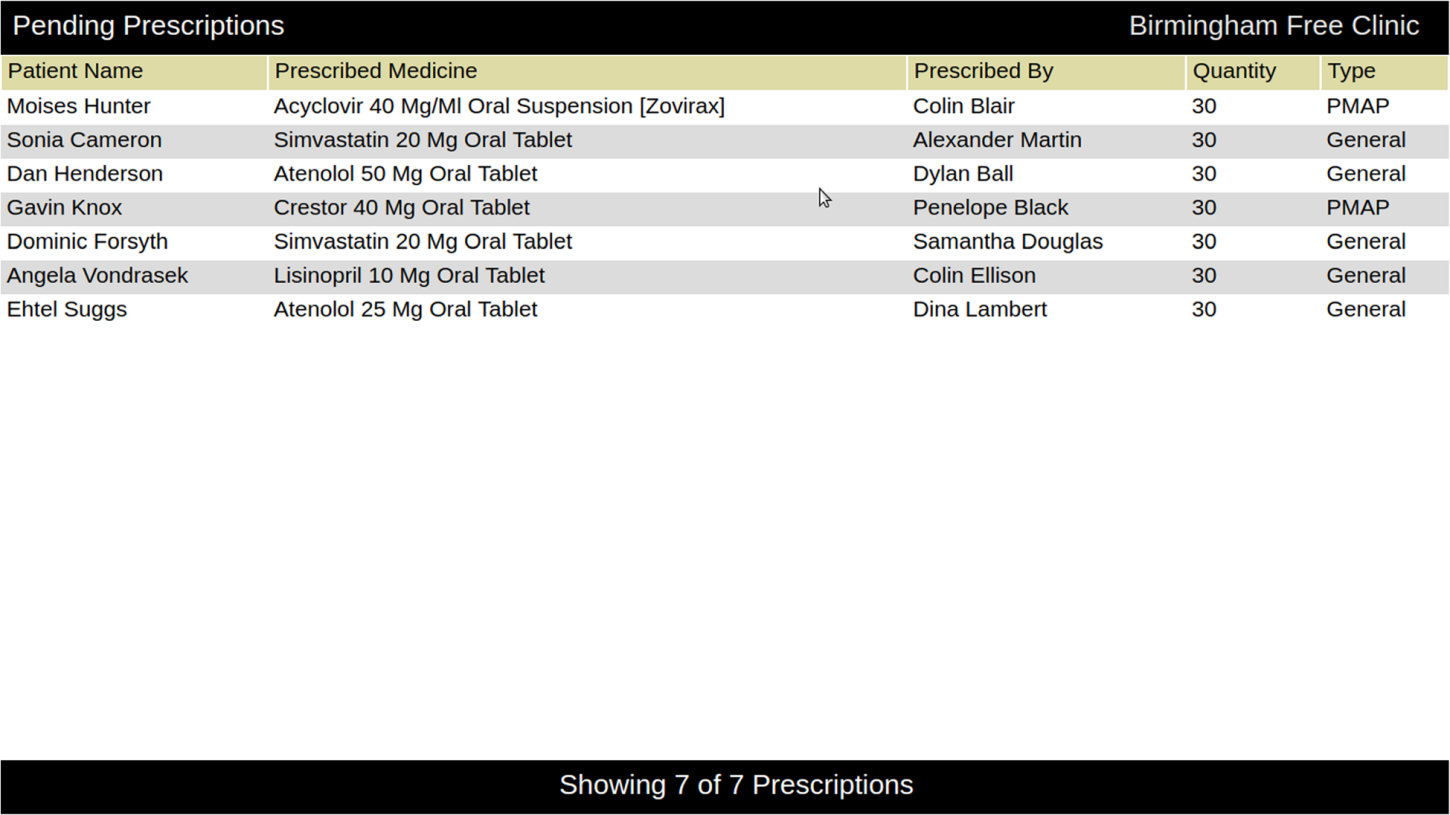
Prescription dashboard. Prescriptions appear on this wall-mounted dashboard as they are received from the EHR. The patient information shown here is mock data

Incoming prescriptions are added to a screen within the application and are prioritized based on their receipt. In addition to this screen, we provided a wall-mounted dashboard that lists all unfilled prescription orders and alerts pharmacists of new orders as shown in Fig. 2. This dashboard comprises a 23-in. screen and a Raspberry Pi mini-computer.

#### Electronic dispensing

**Fig. 3:**
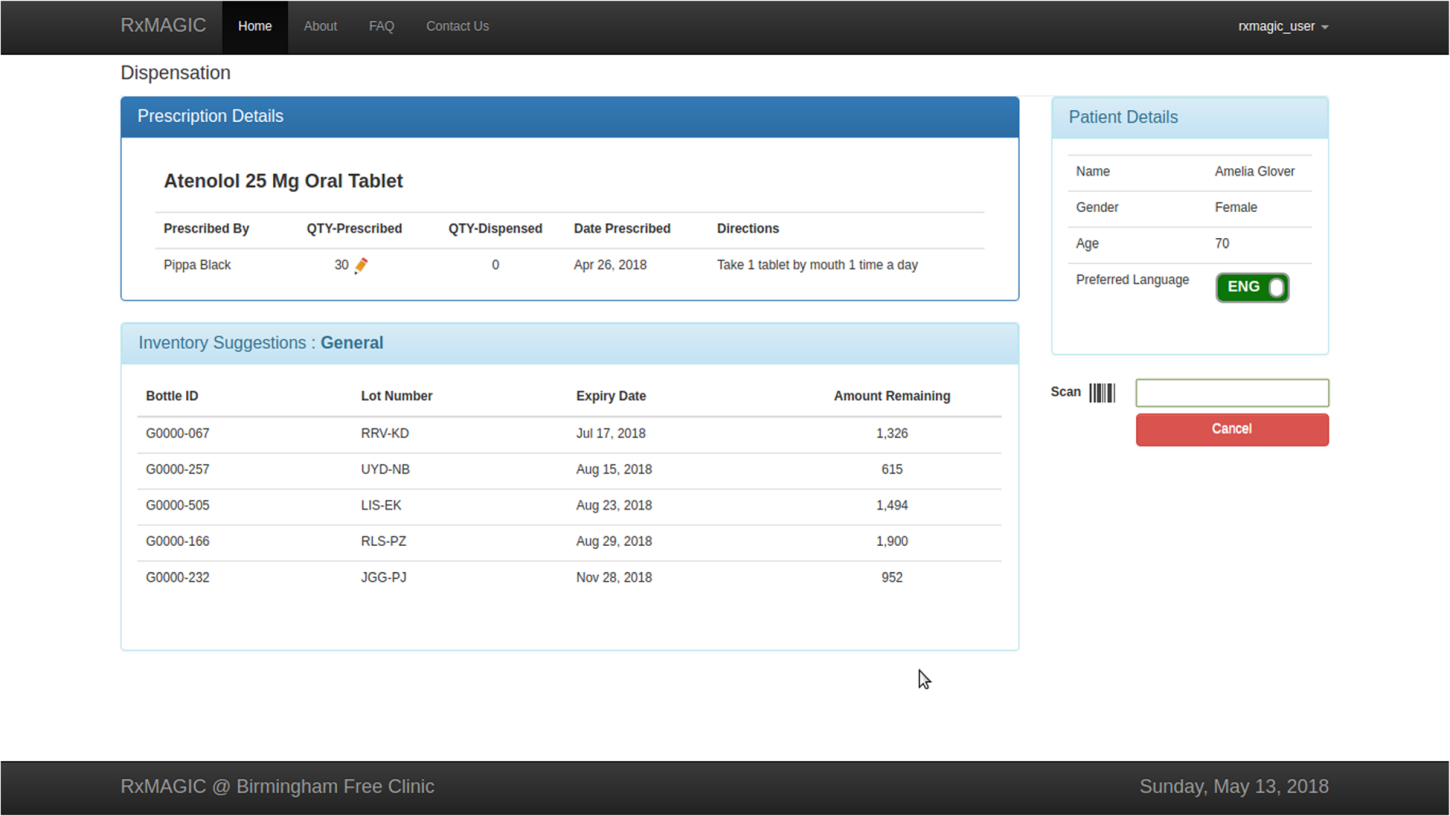
Electronic dispensing view. Users are brought to this view after selecting a prescription on the previous screen. Completion of the dispensing workflow results in a prescription label

Pharmacists dispense medication by selecting a prescription from the prescription screen, which brings them to the patient-specific dispensing screen (Fig. 3). This screen displays prescription and patient demographic information. Pharmacists can modify the prescription quantity and select language preference (English or Spanish), which will dictate how the medication directions are printed; however, at this time, pharmacists are required to translate directions as needed in Spanish.

The dispensing screen provides inventory suggestions based on expiration date and inventory type (i.e. PAP or general inventory). Items closest to expiration will appear at the top of the list to encourage dispensation and avoid drug wastage. To initiate the dispensing workflow, a user must scan the unique barcode label on the medication and enter the dispensed quantity. A computer-generated label is automatically printed once the dispensed quantity equals the prescribed quantity, and the inventory level is adjusted appropriately.

#### Alerting and reporting

**Fig. 4:**
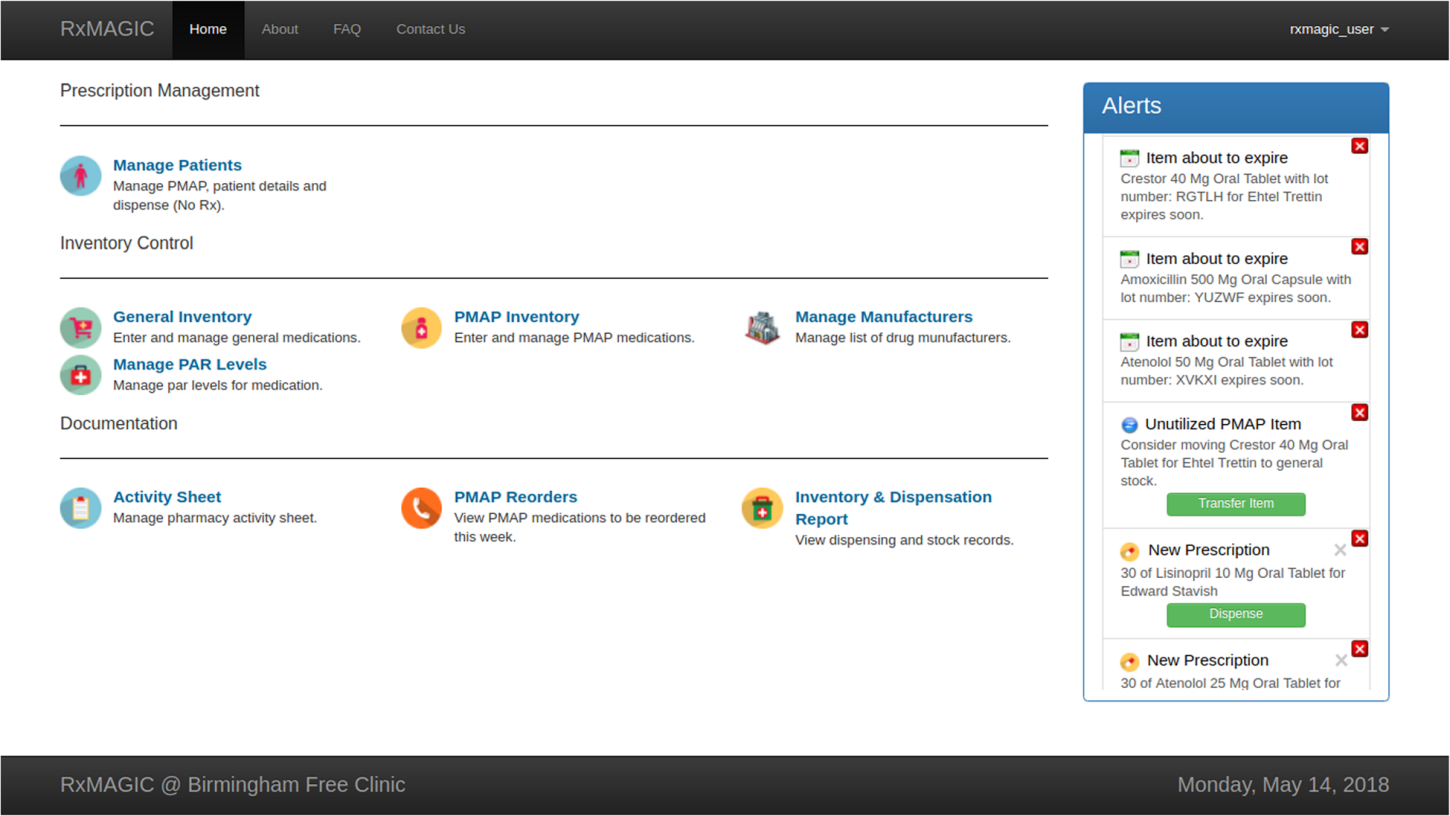
RxMAGIC home dashboard. Users can select actions from this dashboard upon login. The alert-feed on the right is updated in real time

RxMAGIC provides additional functionality apart from inventory tracking and dispensing. An ‘alert feed’ on the home page provides real-time access to alerts that were designed based on the relevant needs of the pharmacist (Fig. 4). These actionable alerts describe items approaching expiration, underutilized PAP items, and low-inventory items. Alerts for underutilized PAP items are generated when a PAP patient has not visited the clinic in six months to alert the pharmacist to contact the patient and/or cancel the PAP.

Alerts for low-inventory items represent the same information that is displayed in the stock summary panel described above. However, the alert-view provides pharmacists with pre-determined courses of action that allow the pharmacist to address alerts in real-time. For example, pharmacists can choose to add the low-inventory item to an electronic report used to facilitate medication reordering. This electronic report replaced a paper document that, in addition to low-inventory items, lists all dispensations for a given clinic session. The reports are saved and viewed in RxMAGIC but can also be exported as PDFs or Word documents.

### Usability study

Two users (i.e., pharmacy students) piloted the study protocol and data collection tools. The pilot study led us to adjust several of the task descriptions to ensure clarity, in addition to building more context into the tasks to create representative scenarios.

Upon completion of the pilot study, six testers were recruited from the BFC to perform the usability testing of RxMAGIC in a laboratory setting. Participants included four pharmacists actively volunteering at the BFC and two AmeriCorps members, which is a representative sample of the total volunteer population at the clinic. The study took place in May 2016 in an office setting outside of the BFC.

Overall, five of the six testers were able to complete each task with no major failures. Of the 11 tasks, all averaged a score of four or five on the ease of completion scale, i.e., all participants reported tasks were somewhat easy or very easy to complete. Tasks three and five in Table 1 had the greatest standard deviation, 1.67 and 1.26, respectively. One participant was unable to complete task three due to a complication with the printer that was resolved immediately. Task five caused confusion for two participants as they were unable to identify the correct workflow within the application to complete the task. However, the participants concluded that challenges with this task could be mitigated with careful training. A high-level description of the 11 tasks and their mean scores on a five-point Likert scale are summarized in Table 1; the actual tasks used in the study provided more situational context.

**Table 1:** Usability tasks and their respective mean scores and standard deviations (SD) for six participants

Task Description		Mean (SD)
1.	Enter medication into general inventory.	4 (0.63)
2.	Edit a previous inventory entry and reprint label.	5 (0)
3.	Dispense medication to patient with prescription.	4 (1.67)
4.	Dispense medication to patient with prescription from multiple bottles, taking their expiration date into account.	4 (0.89)
5.	Dispense medication to patient without a prescription.	4 (1.26)
6.	Identify medications approaching expiration.	4.3 (0.82)
7.	Select an under-stocked medication to add to the electronic stock report used for reordering.	4.8 (0.41)
8.	Describe a patient’s dispensation history at the clinic.	4.8 (0.41)
9.	Adjust the inventory par level for a highly used drug.	4.5 (0.84)
10.	Manage and respond to an alert on the feed.	4.7 (0.52)
11.	Facilitate PAP medication reordering.	4.3 (0.82)

The testers had mostly positive remarks regarding the simplicity of the system, its high learnability, and how closely it mimics their current workflow**.** They were generally enthusiastic about deployment and were able to articulate the potential benefits and efficiencies the system would provide. Direct quotes from the session transcripts include:

*“It’s [RxMAGIC] really modern, it’s very easy to use, like it reminds me of my Gmail inbox. It’s very intuitive, just with the look of how it is. I know what to expect with each click.”*

*“All of this was really easy, it’s very simple. I think somebody new would have no problem, actually, I don’t know if you’d even need to train new people.”*

*“Wow, this would’ve saved me a lot of time last week! Not only that, it just makes me feel safer, like I’m doing a better job.”*

There were a few commonly reported concerns associated with system functionality, such as the frequency and organization of alerts and the information included on medication labels. Further, pharmacists were concerned about the coverage of RxNorm for the medication formulary used at BFC which includes non-prescription items such as syringes, diabetic testing supplies, and multivitamins. Another concern that was raised was the impact RxMAGIC would have on the clinic workflow. Pharmacists recognized that their workflow would change following system deployment but were unsure how that change would affect time utilization and communication between the clinical and pharmaceutical service.

### System deployment

RxMAGIC was deployed in the BFC and the off-site clinic administration office in October 2016 over a two-week period. All computers are located within the UPMC network. The RxMAGIC application itself is hosted on a web server that is physically located in the BFC, whereas the database is hosted on a virtual server in the UPMC data center. This was done to adhere to security requirements within the UPMC enterprise, however, other configurations are possible depending on the security requirements of the organization.

## Discussion

In this manuscript, we describe the user-centered design process and deployment of a novel dispensary management information system in a free clinic. Components of RxMAGIC are described in the context of the BFC workflow, which is the first implementation site of this system. We encountered few challenges during system deployment that were mostly related to the EHR integration and our use of standards, none of which required us to stop system use. Within the weeks following deployment, we made several modifications to our handling of HL7 messages and the translation of NDC identifiers to RxNorm terms, which significantly improved integration and data sharing. We consider the deployment to be successful in that users reported high learnability and a seamless integration into their existing workflow. The paper-based system was phased out within a month of system use. RxMAGIC has been used daily since its deployment in October 2016.

The process used to design RxMAGIC was atypical in that our initial goal was not to develop and deploy a sophisticated system. Instead, we planned to implement a series of informatics interventions tightly coupled to specific user needs and challenges identified in the dispensing workflow at the BFC. This problem-centric approach is beneficial in that unnecessary functionality will not diminish the user experience and reduce the utility of the system. Further, the purpose of the intervention and the outcome for which it optimizes can be clearly articulated and measured.

While this notion of a medication management information technology is not a new concept, a literature search revealed that little is known regarding their use and impact in free and charitable clinics. RxMAGIC is novel in that it may be the first open-source, stand-alone, interoperable dispensary management information system designed to support the unique challenges associated with medication management as typified by the BFC, such as the ability to support the procurement and distribution of multiple inventories. Further, as RxMAGIC is an open-source project, it has minimal costs associated with its implementation and maintenance, making it a sustainable option for low-resource clinics.

In order to be successful, an independent system such as RxMAGIC must overcome several barriers to promote adoption in this setting. A critical barrier is a system’s inability to exchange healthcare data with existing technologies in an enterprise. While this may be hindered by bureaucratic challenges, the use of health data standards is necessary to achieve interoperability and a seamless integration [14]. Thus, HL7 messaging and RxNorm play a significant role in RxMAGIC’s successful adoption at the BFC. These standards were chosen due to their existing implementation in the free clinic, in addition to ensuring generalizability to future sites. Further, enabling users to login to RxMAGIC with their UPMC credentials provides added value and convenience for the clinical users.

Some research groups have developed technological solutions for free clinic dispensaries, however their inability to interoperate hindered their generalizability. Rosenbaum et al. developed a pharmaceutical tracking system for a free clinic that utilizes RxNorm to support the acquisition and management of medications. While this system has improved inventory management, it does not provide support for the entire dispensing process nor is it capable of interoperating with an EHR [17]. Similarly, AmeriCares, a non-profit global health organization, has recently piloted an inventory management program for selected free clinics in the US [18]. This software offers support for many components of the medication management continuum. However, this system does not use standard vocabularies, which can lead to challenges with interoperability. This, in conjunction with a significant implementation fee, may lead to implementation failure at some clinics.

We understand that free clinic workflows may vary between sites, however colleagues have indicated similar challenges to those documented at the BFC. Therefore, although RxMAGIC was developed in the context of the BFC, we believe components are generalizable to other low resource clinics. For example, it is likely that a free clinic may not have an EHR or any system to support order entry. While RxMAGIC provides value in that it can exchange data with other systems, we have implemented a feature that enables electronic dispensation should orders fail to transfer electronically. Thus, if a clinic does not have an EHR or an electronic prescribing framework, the dispensing and inventory components can still be utilized in RxMAGIC. We plan to test this notion of generalizability through implementing aspects of RxMAGIC on touch-screen clinical workstation appliances in a primary care clinic in Honduras.

Our usability testing uncovered user concerns regarding non-prescription items that are not currently included in RxNorm, such as syringes, diabetic testing supplies, and multivitamins. These items cannot be managed in RxMAGIC’s inventory because they are not represented as concepts in RxNorm. However, the benefits of using a standard drug-naming database to achieve communicate with the EHR outweighed the challenge associated with RxNorm coverage. We recognize that understanding this challenge related to RxNorm coverage was a limitation of our usability study, as the tasks were carefully constructed to only include RxNorm concepts.

Further, participants also had concerns on the impact RxMAGIC would have on the clinic workflow. For example, as handwriting medication labels could lead to increased patient wait times, pharmacists created a work-around by preparing most medication refills before the medication order had been placed in the EHR. Now, CPOE initiates the dispensing process, which requires physicians to promptly enter medication orders immediately after patient consultation. If physicians delay order entry after the completion of the consultation, patients wait unnecessarily for medicines creating a bottleneck in the dispensation process. While workflow changes such as these are inevitable, they could not be fully understood and resolved until after the system was deployed and in steady use. As the usability evaluation was completed in a controlled environment, we were unable to understand the interoperability component and the effects that may have on the prescribing and dispensing practices. This was a limitation of the usability study. Thus, in future work, we plan to study the impact of RxMAGIC in situ on pharmacist time utilization and satisfaction.

## Conclusions

We built RxMAGIC, a dispensary management information system that augments the pharmacist’s ability to efficiently deliver medication services in a free clinic setting. RxMAGIC is a pharmacist-facing application that provides electronic dispensing, automated inventory management, and enhances process efficiency and transparency across all aspects of care. We leveraged health data standards to deploy RxMAGIC at the Birmingham Free Clinic so that it is capable of communicating with the existing EHR infrastructure. RxMAGIC has been in continued use since its deployment in October 2016.

## Additional file

Additional file 1: Complete list of user stories grouped into themes and sorted by user group as used for RxMAGIC implementation. The 45 user stories are organized into feature-based themes. Following each theme is the number of user stories that comprise it in parenthesis. Each story should be interpreted from the perspective of the identified user group. (PDF 118 kb)

## Abbreviations

BFC: Birmingham Free Clinic
CPOE: Computerized Physician Order Entry
EHR: Electronic Health Record
HL7: Health-Level-Seven
PAP: Patient Assistance Program
RxMAGIC: Prescription Management And General Inventory Control
UPMC: University of Pittsburgh Medical Center
