# Supplementary material for: User-centered design and usability testing of RxMAGIC: a prescription management and general inventory control system for free clinic dispensaries
Source: BMC Health Serv Res. 2018 Sep 10;18:703. doi: 10.1186/s12913-018-3517-8 (PMC6131751; doi:10.1186/s12913-018-3517-8)
Supplement: Supplementary file 1 — Complete list of user stories grouped into themes and sorted by user group as used for RxMAGIC implementation. The 45 user stories are organized into feature-based themes. Following each theme is the number of user stories that comprise it in parenthesis. Each story should be interpreted from the perspective of the identified user group. (PDF 118 kb) [file 12913_2018_3517_MOESM1_ESM.pdf]

Theme 01: View and maintain inventory through a web-based browser. (10)	
Pharmacist	I want to have visibility into the medication inventory so that I can inform appropriate staff of insufficient inventory and facilitate reordering.
	I want to have visibility into the medication inventory so that I don't waste time physically searching for medications in the cabinets that we don't have in stock.
	I want to have visibility into the specific inventories maintained by the dispensary so that I know which cabinet to access when consulted about stock availability.
	I want to enter new medication items into the inventory so that I can attach a unique inventory ID barcode to all stock medications.
	I want to be able to edit drug counts in the inventory so that I can ensure they are accurate at all times.
	I want to be able to reprint inventory ID labels so that each medication item has a legible barcoded label (should one become detached).
	I want to be able to delete items from the inventory so that I can cleanse the inventory of expired medication.
Physician	I want to have visibility into the current formulary during patient visits so that I can have an informed discussion with the patient about their treatment plan without interrupting the pharmacist.
	I want to know if a patient is enrolled in a PAP program so that I know I can prescribe certain types of medications.
AmeriCorp	I want to enter items into the inventory from my own office so that I can deliver them to the clinic labeled and reduce the pharmacist workload.

Theme 02: Use electronic dispensation to update drug counts in real time. (6)	
Pharmacist	I want electronic dispensation to update drug counts in real time so that stock counts are updated accurately and immediately.
	I want electronic dispensation to update drug counts in real time so that I am informed of low-inventory levels in the timeliest fashion.
	I want to be able to electronically dispense medication without a medication order from the EHR so that I can maintain accurate drug counts for OTC medication too.
	I want to know the history of a dispensation for a certain patient (medications patients are prescribed and taking) from the BFC so that I can avoid drug-drug interactions during dispensation.
	I want to understand specific stock availability at the point of dispensation so that I ensure we prioritize dispensation of medications approaching expiry.

Physician	I want to know the history of dispensation for a certain patient from the BFC so that I can prevent an adverse drug event during ordering.
**Theme 03: Produce computer generated labels upon dispensation. (3)**	
Pharmacist	I want to attach preprinted adhesive labels to dispensed medication so that my documentation tasks become more efficient.
	I want to attach preprinted adhesive labels to dispensed medication so that the patient clearly understands the medication directions throughout the duration of the prescription.
	I want to attach preprinted adhesive to prescription medication so that they are complete with all necessary information required for adherence to dispensing standards.
**Theme 04: Automatically alert pharmacists of new medication orders after CPOE. (6)**	
Pharmacist	I want to be alerted immediately when a physician has entered medication orders so that we can efficiently initiate the dispensing process and reduce patient wait times.
	I want to be alerted immediately when a physician has entered medication orders so that I can ensure that the drug is in stock prior to the end of the patent visit.
	I want to know when a physician is confused during CPOE so that we can resolve the problem and correctly update the order in EpicCare.
	I want incoming prescriptions to be automatically added to a list in order of their receipt so that I prioritize dispensation to the longest-waiting patients.
	I want incoming prescriptions to be automatically added to a visible dashboard so that I do not need to continually check the patient dashboard in EpicCare.
Physician	I want the pharmacist to be alerted immediately when I enter a medication via CPOE so that patients aren't waiting for long periods of time if I forget to update their status.
**Theme 05: Provide soft-stop alert feed functionality that is updated in real time. (6)**	
Pharmacist	I want to know the expiration date of all medications so that I can prioritize dispensation of near-expiry drugs to reduce wastage.
	I want to know when medications are expired so that I can keep patients safe by deleting them from the inventory.
	I want to know the lot number of all medications so that I can quickly respond to medication recalls and keep patients safe.
	I want to customize par levels so that I am in control of low inventory alerts.
	I want to be notified when a medication item has fallen below a given threshold so that I can promptly add the item to the order sheet to avoid stockouts.

	I want to know when a PAP patient hasn't returned to the clinic in six months so that I can call the patient or cancel their application
**Theme 06: Automatically generate the activity sheet and enable PDF creation. (8)**	
Pharmacist	I want electronic dispensation to populate the activity sheet so that my documentation tasks are more efficient.
	I want to be able to search and view all old activity sheets within RxMAGIC so that I understand consumption patterns.
	I want to be able to print the activity sheet so that it gets to delivered to the PHCUP.
	I want to know when a patient has one month or less of a PMAP medication in stock so that I can indicate its due for reorder on the activity sheet.
	I want low inventory medications to be automatically added to the activity sheet so that I don't have to remember low-stock items throughout the entire clinic session.
AmeriCorp	I want to view an automated copy of the activity sheet daily so that I know it is accurate, legible, and on time.
	I want to view an automated copy of the activity sheet so that I know which medications to reorder.
	I want to be able to export and print the activity sheet so that I can save it for my own records.
**Theme 07: Provide medication reordering support for the PAP application process. (5)**	
Pharmacist	I want to know the details of a patient's PAP application so that I can ensure their medication is up-to-date and reordered.
	I want to ensure a patient is enrolled in a PAP application before adding their medication to the inventory.
AmeriCorp	I want to know the 'date to reorder' a patient's medication so that I can ensure an uninterrupted supply of PAP medications.
	I want to view a report of all medications to be reordered in a certain time period so that I don't forget to reorder a patient's medication.
	I want to know the specific pharmaceutical company program in which a patient is enrolled so that I know who to contact for reordering.
**Theme 08: Establish a user management framework. (1)**	
All	I want to be able to login to RxMAGIC with my UPMC credentials so that I don't need to create another set of credentials.
